# Harsh parenting among veterans: parents' military-related PTSD, mentalization, and pre-military trauma

**DOI:** 10.3389/fpsyg.2023.1283801

**Published:** 2023-12-19

**Authors:** Xiafei Wang, Qingyang Liu, Gabriel J. Merrin, Amanda Keller, Dalhee Yoon, Ava Henderson

**Affiliations:** ^1^School of Social Work, David B. Falk College of Sport and Human Dynamics, Syracuse University, Syracuse, NY, United States; ^2^Department of Human Development and Family Science, David B. Falk College of Sport and Human Dynamics, Syracuse University, Syracuse, NY, United States; ^3^Department of Social Work, McGill University, Montreal, QC, Canada; ^4^Department of Social Work, Binghamton University-State University of New York, Binghamton, NY, United States

**Keywords:** veterans, posttraumatic stress disorder, harsh parenting, pre-mentalization, pre-military trauma

## Abstract

**Objectives:**

Veteran parents experiencing posttraumatic stress disorder (PTSD) may resort to harsh parenting. The indirect pathway from parental military-related PTSD to harsh parenting, and the moderating role of parents' pre-military trauma histories, has been less explored. Informed by mentalization theory, as well as trauma-sensitive and posttraumatic growth perspectives, we aim to explore the associations between veteran parents' military-related PTSD, mentalization, harsh parenting, and prior trauma before military service.

**Methods:**

Data were collected from an online research panel of 509 veteran parents with children under 10. We employed Structural Equation Models to test indirect and moderating effects.

**Results:**

We identified an indirect effect of parental pre-mentalization from military PTSD to harsh parenting [corporal punishment: *b* = 0.35, *p* < 0.001, 95% CI (0.23, 0.46); psychological aggression: *b* = 0.14, *p* < 0.001, 95% CI (0.09, 0.19)]. Multi-group analysis on four parent groups (parents with only pre-military physical trauma, parents with only pre-military psychological trauma, parents with both pre-military physical and psychological trauma, and parents with no pre-military physical or psychological trauma) highlighted differences in these associations, particularly between parents with only pre-military physical trauma and those without any physical and psychological trauma. The military-related PTSD effects on psychological aggression, corporal punishment, and pre-mentalization were all significantly higher for parents without pre-military physical and psychological trauma.

**Conclusion:**

Modifying parents' interpretation of their child's mental states can potentially counteract the effects of veterans' military PTSD on harsh parenting. Family-based programs should be created considering veteran parents' pre-military trauma histories.

## Introduction

Veterans frequently suffer from posttraumatic stress disorder (PTSD; Creech and Misca, 2017). PTSD is a traumatic stress reaction to “actual or threatened death, serious injury, or sexual violence” (DSM-5, American Psychiatric Association, 2013, p. 271), including symptoms of (1) intrusion; (2) the presence of persistent avoidance of stimuli associated with the trauma; (3) negative alterations in cognitions and mood associated with the traumatic event(s); (4) marked alterations in arousal and reactivity as it relates to the trauma (DSM-5, American Psychiatric Association, 2013, p. 271). Due to their combat experience, veterans are often exposed to actual or threatened death and serious injuries, making them more susceptible to experiencing PTSD. Data from the US Department of Veterans Affairs (VA) indicates that veterans are diagnosed with PTSD at a higher rate than the general population. Specifically, 23% of veterans from Operation Enduring Freedom (OEF) and Operation Iraqi Freedom (OIF) have been diagnosed with PTSD, a rate approximately four times higher than the general population (Creech and Misca, 2017). This elevated PTSD prevalence increases veteran parents' likelihood of adopting harsh parenting behaviors (Christie et al., 2019). Considering nearly two-fifths of U.S. military personnel are parents (Creech and Misca, 2017), the repercussions of PTSD potentially extend beyond veterans to their children. Consequently, these children might encounter adverse environments that can detrimentally affect their socio-emotional development (Creech and Misca, 2017).

Parental mentalization, the appropriate interpretation of their child's internal states, fosters parenting sensitivity and encourages positive parenting practices (Cohen et al., 2022). On the other hand, compromised mentalization is associated with distorted sense of identity, which results in a reduced sense of responsibility for one's own behaviors. People with compromised mentalization may treat others as physical objects, and then disregard the psychological effects of their brutal behaviors on others (Fonagy et al., 1997).

However, traumatic symptoms can significantly hinder a parent's ability to mentalize (Allen et al., 2012; Janssen et al., 2022). Notably, parents with PTSD might adopt a pre-mentalization mode, a distorted way of interpreting their child's subjectivity as malevolent, potentially leading to harsh parenting or child maltreatment (Edler et al., 2022). Further complicating matters, veterans often confront with additional challenges. Those with military backgrounds have higher incidences of pre-military trauma, such as experiencing physical punishment from their own caregivers and emotional mistreatment (Blosnich et al., 2014). Consequently, veteran parents face the compounded effects of military-related PTSD and pre-military trauma, which may complicate the association between their PTSD and harsh parenting.

To develop effective prevention and intervention for children in veteran families facing the risk of harsh parenting, it is essential to understand the underlying mechanisms connecting military-related PTSD to harsh parenting and how pre-military trauma histories might influence these pathways. Thus, this study aims to first investigate the indirect effect of parental pre-mentalization between the association of veteran parents' military-related PTSD and harsh parenting. Given the well-established direct effect of veterans' PTSD on harsh parenting in existing literature, our study shifts focus to the indirect effect of parental pre-mentalization. We focus on the indirect effect of parental pre-mentalization due to its relatively limited research coverage and its higher potential for modification through psychotherapies; Second, we aim to determine if traumatic experiences of veteran parents prior to military service moderate the associations between parental military-related PTSD, pre-mentalization, and harsh parenting.

### PTSD and harsh parenting

Parental PTSD significantly predisposes individuals to harsh parenting. Children with parents suffering from PTSD often report increased emotional abuse and neglect, as the trauma hinders parents from validating their children's feelings (Yehuda et al., 2001). Additionally, PTSD symptoms, like heightened arousal and reactivity can lead to over-reactive or harsh parenting (Franz et al., 2022). Christie et al. (2019) posits that PTSD symptoms, including anger, reduced affect, and attention and memory impairments, correlate with irritable and aggressive parenting behaviors.

Veterans' distinct experiences amplify the importance of understanding how PTSD symptoms influence parenting (Creech and Misca, 2017). Their worldviews shaped by military-related PTSD can disrupt foundational aspects of trust, intimacy, power, and control, which in turn, influence the parent-child relationship (Hopson, 2017). Research on veterans with PTSD indicates reduced parenting satisfaction, elevated parenting stress, increased negative parenting behaviors, challenges in managing anger, and potential escalation to violent actions (Christie et al., 2019). As studies on parenting delve deeper into the relationship between parental PTSD and harsh parenting, it's crucial to pinpoint the underlying mechanisms that lead to this phenomenon.

### PTSD, mentalization, and harsh parenting

One connection between PTSD and aggressive behavior is deficits in social cognition (Nietlisbach and Maercker, 2009; Sharp et al., 2012), which lead to the concept of “mentalization.” Mentalization, also operationalized as “reflective functioning,” refers to a person's capacity to perceive human behavior as driven by mental states, such as thoughts, feelings, desires, and beliefs (Fonagy et al., 2018). Effective mentalization enables individuals to predict and influence others' behaviors, fostering successful social interactions (Sharp et al., 2012). Those with good mentalizing capacity view others as separate beings with unique mental states, and they will actively seek to understand these states.

Research suggests a connection between PTSD and compromised mentalization (Allen et al., 2012; Janssen et al., 2022). Individuals with PTSD symptoms may struggle with their mentalizing capacity, particularly discerning between inner emotions and external realities, as well as differentiating their inner mental states from those of others. For example, flashbacks and avoidant symptoms can blur the lines between imagination and reality and between genuine dangers and feelings of fear (Palgi et al., 2014). Hyperarousal symptoms might prompt an individual to misinterpret ambiguous situations as threats and assume negative intentions in others (Taft et al., 2008). Also, neuroscientific evidence shows that certain brain regions vital for mentalization, especially the medial frontal lobes, appear to be compromised in those with PTSD (Frith and Frith, 2003; Sharot et al., 2007).

In the context of parenting, parental mentalization denotes “a parent's capacity to represent and understand the breadth of his/her child's internal experience” (Slade, 2005, p. 275). Luyten et al. (2017) specified three specific dimensions of impaired parental mentalization: (1) pre-mentalizing, (2) over certainty, and (3) lack of genuine interest and curiosity. Pre-mentalizing refers to parents' interpreting their child's mental states in a distorted manner, often perceiving them as malevolent. Over certainty is defined as parents' limited awareness of the opacity of their child's mental states, resulting in an intrusive or presumptuous understanding. Parents' lack of genuine interest and curiosity refers to parents' minimal interest in exploring their children's mental states.

Parental mentalization is strongly associated with parenting practice (Smaling et al., 2016a,b; Yule, 2021; Cohen et al., 2022; Edler et al., 2022). Higher levels of parental mentalization were associated with reduced parents' insensitivity (Smaling et al., 2016a) and decreased physical aggression (Smaling et al., 2016b). During the COVID-19 pandemic, Cohen et al. (2022) found that parents exhibiting greater mentalization demonstrated positive parenting behaviors, even amidst parental distress caused by the pandemic. Conversely, parental pre-mentalization, characterized by a distorted way of interpreting their child's subjectivity as malevolent (Luyten et al., 2017), has been linked to less supportive reactions to children's emotions (Edler et al., 2022) and a decrease in emotionally validating parenting behaviors (Yule, 2021).

While the links between PTSD and compromised mentalization, as well as between pre-mentalization and harsh parenting, are well documented, these dynamics are underexplored in parents with military backgrounds. In studies that focus on military individuals, connections between PTSD and mentalization often appear inconclusive, possibly due to limited sample sizes (Mazza et al., 2012). Given that pre-mentalization signifies a severely diminished mentalizing ability (Burkhart et al., 2017) and established research links it to detrimental parenting behaviors (Yule, 2021; Edler et al., 2022), our study aims to elucidate the relationship between military-related PTSD, pre-mentalization, and harsh parenting practices.

### Parents' pre-military trauma as a potential moderator

While military-related PTSD stands out as a prominent concern for parents with military backgrounds, it's crucial to recognize that these parents, like everyone else, might suffer from trauma before their military services. Analysis from the 2010 Behavioral Risk Factor Surveillance System reveals that individuals with military backgrounds have a heightened susceptibility to adverse childhood experiences (ACEs) such as being maltreated by their family members (Blosnich et al., 2014). Some might turn to the military as refuge from turbulent household environment. Given the disproportionately high rates of ACEs among military personnel, questions arise about how these pre-military traumatic experiences may compound with military-related PTSD, further influencing the wellbeing of their children.

There are two prevailing frameworks offering divergent theoretical perspectives on the moderating role of veteran parents' pre-military trauma. From the trauma-sensitive viewpoint, trauma can heighten a survivor's susceptibility to psychological distress and vulnerability in the face of subsequent traumatic events (Selye, 1976; Solomon, 1993). Thus, bearing prior traumatic histories before joining in the military might increase the negative repercussions of parents' military-related PTSD. Following this reasoning, the risks associated with pre-mentalization and harsh parenting could intensify among parents who have faced military-related PTSD if they have also encountered traumas before their military service, such as pre-military physical or psychological trauma. Furthermore, varying types of past trauma might influence reactions to PTSD differently. For instance, research on women who have faced partner violence indicates that psychological abuse has a more profound association with PTSD symptoms compared to those who have only faced physical abuse (Taft et al., 2005).

In contrast to the trauma-sensitive perspective, the posttraumatic growth framework posits that individuals can surpass their pre-trauma levels of adaptation, psychological functioning, or life awareness in the aftermath of trauma (Tedeschi et al., 1998). Posttraumatic growth manifests across three domains: self-perception, interpersonal relationships, and life philosophy (Tedeschi and Calhoun, 1995). Several studies have shown that individuals with a history of trauma often report a fortified sense of self, a renewed appreciation for life, and the development of adaptive coping mechanisms (Shakespeare-Finch and De Dassel, 2009; Wang et al., 2019), which may act as buffers against negative behaviors, such as harsh parenting. It's noteworthy that posttraumatic growth is prevalent among U.S. veterans. For instance, amidst the COVID-19 pandemic, many veterans reported experiencing pandemic-induced posttraumatic growth, such as heightened appreciation of life, improved interpersonal relationships, and bolstered personal resilience (Pietrzak et al., 2021). The way an individual perceives the influence of prior trauma on their personal development is integral in fostering posttraumatic growth. Drawing from a study on ex-prisoners-of-war (POWs) during the pandemic, those who viewed their prior war-related traumas as instrumental in shaping their coping mechanisms exhibited lower PTSD incidences compared to those who deemed such experiences detrimental (Solomon et al., 2021). Thus, pre-military trauma might not inevitably escalate the risks of pre-mentalization and harsh parenting for veteran parents living with PTSD.

The absence of prior research examining the interactive effects of veteran parents' pre-military trauma and military-related PTSD on their parenting underscores the significance of this study. Investigating how pre-military trauma moderates the relationship between military-related PTSD, pre-mentalization, and harsh parenting will broaden our understanding on veteran families and contribute novel insights to the field.

### The current study

**Figure 1:**
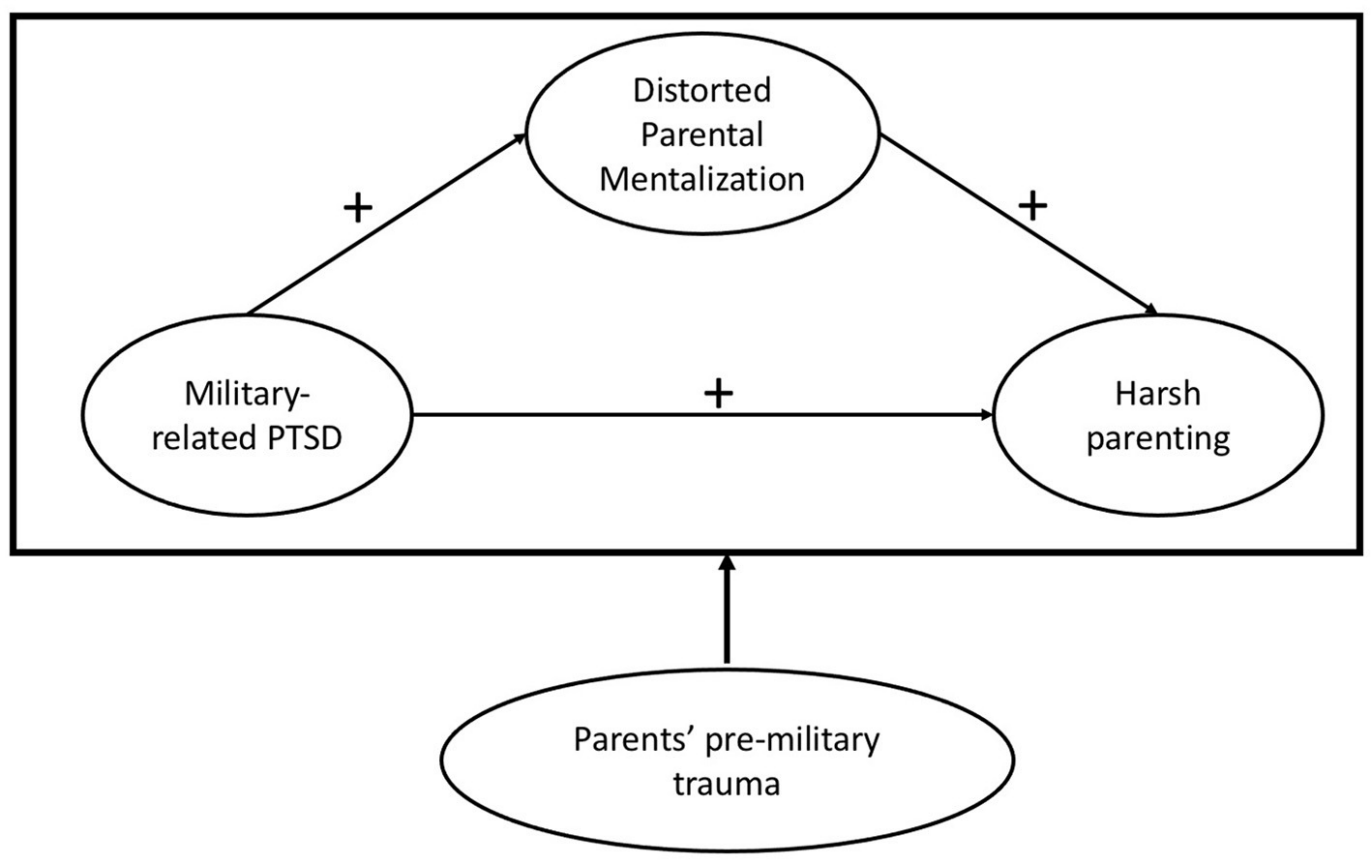
Conceptual model.

Based on the theoretical propositions about mentalization (Sharp et al., 2012), we hypothesized that higher levels of military-related PTSD symptoms would be linked to higher levels of pre-mentalization in veteran parents. Sequentially, higher levels of pre-mentalization would be associated with harsh parenting. Informed by the trauma-sensitive and posttraumatic growth theories, we would examine whether those associations vary by parents' pre-military trauma. According to trauma sensitive theory, the associations between military-related PTSD, pre-mentalization, and harsh parenting would be stronger for parents with a history of pre-military trauma compared to those without such history. According to posttraumatic growth theory, those associations would be weaker for parents with a history of pre-military trauma, suggesting an individual's potential for growth after trauma (Figure 1: Conceptual Model).

Through examining the indirect effect of parental pre-mentalization, our study enhances the understanding of the pathway between PTSD and parenting behaviors. This research also holds implications for preventing child maltreatment, as harsh parenting may escalate to such levels. By incorporating trauma-informed perspectives, we further discern how pre-military trauma in veteran parents moderates the relationship between military-related PTSD, mentalization, and parenting outcomes. By addressing the dual knowledge gap—veteran parents in parenting research and parenting in military studies, our study findings can inform professionals to provide better services for veteran families.

## Materials and methods

### Participants and procedure

Upon securing approval from the university's institutional review board (IRB), we collaborated with Qualtrics to recruit a purposive sample of veterans parenting children under the age of ten. This focus on younger children was deliberate, as they are more susceptible to harsh parenting compared to their older counterparts. Qualtrics, a renowned technology firm, aids researchers in participant recruitment through its representative online research panel. This panel consists of a curated group of respondents pre-consented to partake in online survey research (for details, refer to https://www.qualtrics.com/research-services/online-sample/). Nowadays, social scientists increasingly use online research panels provided by companies such as Qualtrics, Amazon.com's Mechanical Turk, and Facebook advertisements for survey research due to their efficiency in recruitment, high-quality data, and reduced logistical costs (Dupuis et al., 2013). The advantages of these platforms were particularly pronounced during the pandemic when our study's data collection occurred, because it was hard to reach participants in person. Among various online research panels provided by technology firms, Qualtrics stands out for its demographic and political representativeness (Boas et al., 2020).

For our study's purposes, Qualtrics implemented two specific eligibility criteria for its online research panel: (1) confirmed veteran status and (2) parenting a child currently aged below 10 years. If the potential respondents self-reported that they met these two criteria, they would be screened into the study. Participants completed all questionnaires using Qualtrics' online survey platform. Before starting the survey, they were required to review and consent to a form attached at the beginning. This form included essential details such as a statement that the project is research and participation is voluntary, a brief overview of the study (including its purpose, duration, and procedures), potential risks or discomforts, expected benefits, and information about incentives. The survey started only after participants gave their consent. Each participant received a $10 incentive as a token of appreciation for their time. Participants received the incentives regardless of their answers. This amount was determined in consultation with our university's IRB officer to ensure it was not coercive but merely a modest gesture of gratitude.

In relation to sample size, Kline (2015) recommends a minimum of 200 participants to ensure sufficient power for detecting significant results using structural equation modeling. Balancing fiscal constraints with the pursuit of rigorous research, we aimed for a sample size of approximately 500. Of the 2,349 respondents from the research panel who expressed interest, 509 met the criteria, successfully passed attention checks, and provided complete data.

Table 1 presents the demographics of our participants. Most participants were male (76.2%), with an average age of 39 years. Their children were 60.6% boys and 39.4% girls, averaging 6.5 years old. A majority of our sample identified as White/European American (78.6%). Additionally, 67.6% of participants held a bachelor's degree or higher. The majority (81.1%) were married, and over half of the participants reported a household income exceeding $70,000.

**Table 1:** Sample characteristics (*N* = 509).

	***N***	**%**	***M* (*SD*)**	**Range**
**Child characteristics**				
Age	498		6.5 (2.62)	0–10
Sex (male)	308	60.6		
**Parent/family characteristics**				
Age	508		39 (9.3)	21–76
Gender (male)	387	76.2		
**Race/Ethnicity**				
Native American/Native North American	10	2.0		
Asian and Asian American/Pacific Islander	12	2.4		
Hispanic/Latinx	32	6.3		
Black/African American	43	8.4		
White/European American	400	78.6		
Multiracial	12	2.4		
**Education level**				
High school diploma/GED	33	6.5		
Some college (no degree obtained)	70	13.8		
Associate degree/Trade school	61	12.0		
Bachelor's degree	170	33.4		
Master's degree	152	29.9		
Doctoral/Professional degree	23	4.5		
**Household income**				
< $10,000	4	0.8		
$10,000–39,999	69	13.5		
$40,000–69,999	87	17.1		
$70,000–99,999	125	24.6		
$100,000–149,999	151	29.7		
More than $150,000	73	14.3		
**Relationship status**				
Married	413	81.1		
Divorced	47	9.2		
Separated	8	1.6		
Widowed	8	1.6		
Never married	33	6.5		

### Measurement

#### Parental PTSD

Parental PTSD was measured by a 17-item PTSD Checklist – Military Version (PCL-M; Weathers et al., 1991) commonly used by Veteran Services (VA) for military-related PTSD screening. PCL-M could be used for aiding in diagnostic assessment and monitoring change in PTSD symptoms among veterans. PCL-M mainly assesses four categories of PTSD symptoms based on DSM-IV: re-experiencing, avoidance, negative alteration in cognition & mood, and hyperarousal. Sample questions included “Suddenly acting or feeling as if a stressful military experience were happening again” and “Avoid thinking about or talking about a stressful military experience or avoid having feelings related to it.” Responses were measured by a 5-point Likert scale ranging from 1 (“Not at all”) to 5 (“Extremely”), with a higher score indicating severe PTSD symptoms. Weathers et al. (1993) suggest a threshold score of 50 as ideal for identifying a likely diagnosis of combat-related PTSD. Prior research showed a 0.97 test-retest reliability and 0.89 or above internal consistency coefficients of the PCL-M (Weathers et al., 1993). In this study sample, the reliability α of the PCL-M was 0.97.

#### Parental pre-mentalization

The parental pre-mentalization was assessed by the 6-item pre-mentalization subscale of the Parental Reflective Functioning Questionnaire (PRFQ; Luyten et al., 2017). The scale developer defined pre-mentalization as “a nonmentalizing stance, malevolent attributions, and an inability to enter the subjective world of the child” (Luyten et al., 2017, p. 8). Sample questions include, “The only time I'm certain my child loves me is when he or she is smiling at me.” “My child cries around strangers to embarrass me.” “I find it hard to actively participate in make-believe play with my child.” “My child sometimes gets sick to keep me from doing what I want to do.” “When my child is fussy, he or she does that just to annoy me.” “Often, my child's behavior is too confusing to bother figuring out.” The response ranged from 1 to 7 (1 = “strongly disagree,” 2 = “disagree,” 3 = “somewhat disagree,” 4 = “neither agree nor disagree,” 5 = “somewhat agree,” 6 = “agree,” 7 = “strongly agree”). Parental pre-mentalization sub-scale has been shown a good reliability (α = 0.7) in a study on mothers with children aged 0 to 36 months (Luyten et al., 2017). One validation study suggested good construct validity of parental pre-mentalization as it was negatively associated with parental coping and satisfaction (De Roo et al., 2019). The reliability α of the parental pre-mentalization was 0.97 in this study.

#### Harsh parenting

Harsh parenting was measured by two distinct elements: corporal punishment and psychological aggression. Corporal punishment aims to inflict physical discomfort or pain, without causing harm or injury. Psychological aggression involves actions like shouting at a child or labeling them with derogatory terms like “dumb” or “lazy” (Straus et al., 1998). In this study, corporal punishment and psychological aggression were both assessed by four-item sub-scales of the Dimensions of Discipline Inventory (DDI, Straus and Fauchier, 2007).

Sample questions for corporal punishment were “How often did you spank, slap, smack, or swat this child?” “How often did you use a paddle, hairbrush, belt, or other objects?” Questions measuring psychological aggression included “How often did you shout or yell at this child?” “How often did you try to make this child feel ashamed or guilty?” The responses ranged from 0 to 9 (e.g., 0 = “Never and not in the past year, but in a previous year;” 9 = “Two or more times a day”).

Based on the DDI manual developed by Straus and Fauchier (2007), we recoded the responses of corporal punishment and psychological aggression as 1 = “ever happened in the past year” and 0 = “never happened in the past year.” In the Straus and Fauchier (2007)'s study, the reliability α of the measures of corporal punishment and psychological aggression ranged from 0.74 to 0.81. In our study, they were 0.79 and 0.73, respectively.

#### Parents' pre-military trauma

Parents' pre-military trauma was assessed by two questions from the Deployment Risk and Resilience Inventory-2 designed to assess military members' traumatic life experiences before joining the military (Vogt et al., 2013). Parents' history of physical trauma was measured by a dichotomous item (Yes/No), “I was physically punished by a parent or primary caregiver,” while psychological trauma was also assessed by a dichotomous item (Yes/No), “I was emotionally mistreated.” Using these two questions, we were able to categorize our participants as veteran parents who (1) experienced both pre-military physical and psychological trauma, (2) experienced only pre-military physical trauma only, (3) experienced only pre-military psychological trauma, and (4) had no pre-military physical or psychological trauma.

#### Covariates

As parents' and children's demographic and socioeconomic characteristics would confound parenting behavior (Belsky, 1984), we included covariates of child age and gender, as well as parents' age, gender, race, relationship status, education and family household income. Child and parent's age were continuous variables. Child and parent's gender were recoded as a binary variable (1 = “male,” 0 = “female”) from the original response items (1 = “male,” 2 = “female,” 3 = “transgender male,” 4 = “transgender female,” 5 = nonbinary/gender fluid) because no respondent identified with other gender categories. Parental race (Native American/Native North American, Asian and Asian American/Pacific Island, Hispanic/Latinx, Black/African American, other/multiracial, reference group = White/European American), relationship status (widowed, divorced, separated, never married, reference group = married) were categorical variables, while parental education (1 = “less than high school,” 2 = “high school diploma/GED,” 3 = “some college,” 4 = “associate's degree/trade school,” 5 = “bachelor's degree,” 6 = “master's degree,” 7 = “doctoral/professional degree”) and family household income (1 = “ <$10,000,” 2 = “$10,000–39,999,” 3 = “$40,000–69,999,” 4 = “$70,000–99,999,” 5 = “$100,000–149,999,” 6 = “More than $150,000”) were ordinal variables.

### Analytic plan

Several steps were conducted to examine our research questions. First, we established a good fitting measurement model for the latent variables, first separately and then together. We used several fit indices to assess model fit, including Chi-square, Comparative Fit Indices (CFI), Tucker-Lewis Index (TLI), Root Mean Squared Error of Approximation (RMSEA), and Standardized Root Mean Squared Residual (SRMR) using standard cut-offs as recommended (Hu et al., 1995; Kline, 2015). An RMSEA/SRMR of 0.05 and below and a CFI/TLI of 0.95 and above indicate a good model fit; an RMSEA/SRMR of 0.05–0.08 and a CFI/TLI of 0.90–0.95 indicate an acceptable fit (Hu et al., 1995). After establishing the measurement models, we tested our hypothesized structural model using the entire sample. All models were fit using Mplus 8.7 (Muthén and Muthén, 2017), and the maximum likelihood (ML) estimator was used to handle nonnormality distribution. There was no missing data among the study variables.

Following, we used group analysis to examine the hypothesized structural model for each of the four trauma groups (parents experienced pre-military physical trauma only, experienced pre-military psychological trauma only, experienced both trauma, and no physical or psychological trauma). Prior to fitting our structural model, we examined measurement invariance across each of the four trauma groups. For binary items, we used the weighted least squares means and variance adjusted estimator (WLSMV) to fit latent variables of corporal punishment and psychological aggression. For continuous items, we used maximum likelihood with robust standard errors (MLR) to fit latent variables of Military PTSD and Pre-mentalization. We followed Wu and Estabrook's (2016) recommendation to examine measurement invariance for the latent variables with binary indicators using the WLSMV estimator that suggest fitting a configural, combined metric and scalar, and strict/unique models. For continuous indicators, we used MLR and followed typical specifications that included configural, metric, and scalar invariance models.

We used the Satorra-Bentler Chi-square difference test for models using MLR and the built-in DIFFTEST option in Mplus for models using WLSMV in combination with a change in CFI and RMSEA <0.01 to assess each test of measurement invariance (Svetina et al., 2020). After establishing measurement invariance for all latent variables, we examined the structural model for each of the four groups. We then examined differences in the magnitude of the effects across groups using Wald tests. Formal tests of the indirect effect of pre-mentalization to military-related PTSD to corporal punishment or psychological aggression were examined using Bootstrapping confidence intervals. For the sake of model parsimony, all models controlled for race and income, as other covariates were not significant.

## Results

### Descriptive statistics

The average veteran parents' military-based PTSD symptoms was 40.58 (*SD* = 18.63) on a scale of 17–85, suggesting that, on average, veteran parents experienced a slightly above low level of military-related PTSD. The level of pre-mentalization was 19.05 (*SD* = 9.13) on a scale of 6–42, showing the average level of sample participants “somewhat disagreed” with the statement of pre-mentalization. Finally, corporal punishment was 1.35 (*SD* = 1.48), and psychological aggression was 1.89 (*SD* = 1.45) on a scale of 0–4, showing the sample's low average levels of harsh parenting.

### Measurement model

For the measurement models, a total of four Confirmatory Factor Analysis (CFA) models were fit for each latent variable (Table 2). Initially, the military PTSD measure had 17 items which was quite a lot for a latent variable. To address this, we used parceling to aggregate the items to reflect four substantively meaningful dimensions of PTSD: re-experiencing, avoidance, negative alteration in cognition and mood, and hyperarousal. The four-item parcels reflecting military PTSD symptoms were then used to create the latent variable. The model with the parcels had an acceptable fit, $\chi_{\left(2\right)}^{2}$ = 35.86, *p* < 0.001, CFI = 0.96, TLI = 0.89, SRMR = 0.02, RMSEA = 0.18. The six items reflecting pre-mentalization had a good model fit, $\chi_{\left(9\right)}^{2}$ = 22.04, *p* < 0.001, CFI = 0.99, TLI = 0.98, SRMR = 0.02, RMSEA = 0.05. The four items used to measure corporal punishment had good model fit, $\chi_{\left(2\right)}^{2}$ = 5.54, *p* < 0.001, CFI = 0.99, TLI = 0.99, SRMR = 0.02, RMSEA = 0.06. Lastly, the four items used to measure psychological aggression had good model fit, $\chi_{\left(2\right)}^{2}$ = 1.42, *p* < 0.001, CFI = 1, TLI = 1, SRMR = 0.01, RMSEA = 0.00.

**Table 2:** Fit indices for four measurement model (*N* = 509).

**Models**	**Chi-square (*df*)**	**CFI**	**TLI**	**SRMR**	**RMSEA**	**90% CI**
1–Military PTSD	35.86 (*2*)^***^	0.96	0.89	0.02	0.18	[0.13, 0.24]
2–Pre-mentalization	22.04 (*9*)^***^	0.99	0.98	0.02	0.05	[0.03, 0.08]
3–Corporal punishment	5.54 (*2*)^***^	0.99	0.99	0.02	0.06	[0.00, 0.12]
4–Psychological aggression	1.42 (*2*)^***^	1	1	0.01	0.00	[0.00, 0.08]

CFI, Comparative Fit Index; TLI, Tucker-Lewis Index; RMSEA, Root Mean Squared Error of Approximation; SRMR, Standardized Root Mean Squared Residual. ^***^p < 0.001.

### Structural model

**Figure 2:**
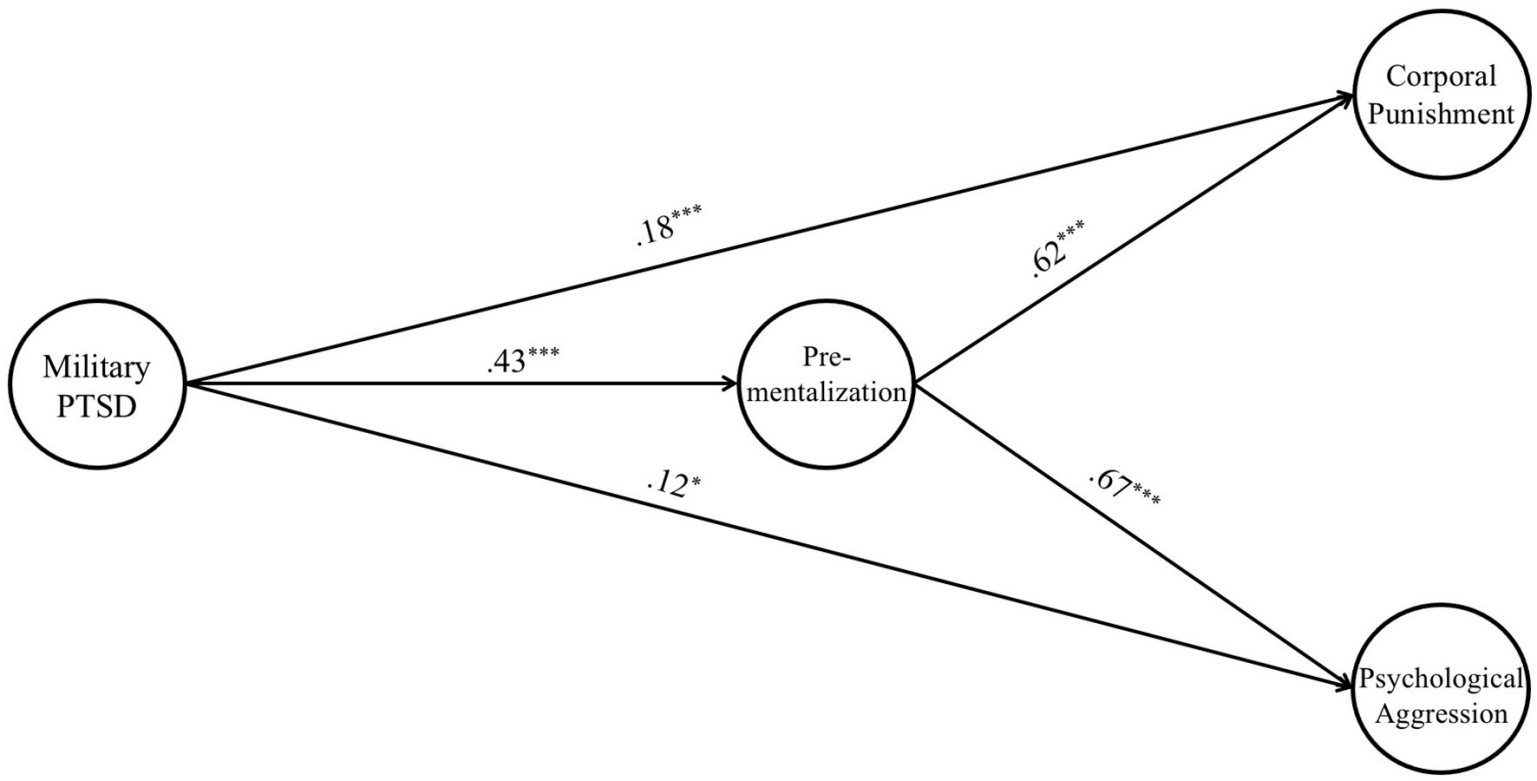
Main effect of veteran's pre-mentalization mediates PTSD toward psychological aggression and corporal punishment (*N* = 509). *X*^2^ = 458.63, df = 157, CFI = 0.90, TLI = 0.88, RMSEA = 0.06, SRMR = 0.09. Report standardized coefficient. Controls parental race and poverty status. Solid line indicates significant and dash line refers to insignificant path. *p < 0.05, ***p < 0.001.

After establishing good fitting measurement models for each latent variable separately and then together, we fit the structural model to examine the main effect of military PTSD, pre-mentalization, corporal punishment, and psychological aggression. The results (see Figure 2) showed that greater levels of military PTSD were associated with the presence of corporal punishment toward children (*b* = 0.18, *p* < 0.001), greater levels of pre-mentalization (*b* = 0.43, *p* < 0.001) and greater levels of psychological aggression (*b* = 0.12, *p* = 0.015). Higher levels of pre-mentalization were positively associated with the presence corporal punishment (*b* = 0.75, *p* < 0.001) and psychological aggression (*b* = 0.72, *p* < 0.001). There were significant indirect effects of pre-mentalization between military PTSD and psychological aggression [*b* = 0.14, S.E. = 0.03, *p* < 0.001, 95% (0.09, 0.19)], and also between military PTSD and corporal punishment [*b* = 0.35, S.E. = 0.06, *p* < 0.001, 95% (0.23, 0.46)].

In terms of covariates, we found that being white was significantly associated with higher levels of pre-mentalization (*b* = 0.15, *p* = 0.001). We also found that above medium income was significantly associated with greater levels of pre-mentalization (*b* = 0.34, *p* < 0.001), corporal punishment (*b* = 0.19, *p* < 0.001), and psychological aggression (*b* = 0.09, *p* = 0.049). The findings on covariates may be explained within the context of the COVID-19 pandemic. Our data was collected in 2021 when vaccines were not widely distributed, a time when individuals still struggled with profound shifts in their lifestyles and accompanying stressors. It is possible that families accustomed to robust support systems, such as being White and rich, may require more adaptation to the challenges of the COVID-19 pandemic, such as the unavailability of daycare, the necessity of sharing spaces due to remote work, and the absence of recreational activities. The more significant the changes in a parent's professional and personal spheres, the greater the stress they could feel, potentially leading to a higher likelihood of exhibiting pre-mentalization and harsh parenting.

### Measurement invariance

To answer the research question that examined differences in our hypothesized structural model across four parent groups with different past trauma, we first established measurement invariance across the groups. We used the MLR estimator for continuous variables and the WLSMV estimator for categorical variables following Wu and Estabrook's (2016) recommendations to examine measurement invariance across groups. These tests of measurement invariance were run in separate steps, given the testing procedures were slightly different (see Table 3). For the continuous variables (i.e., military PTSD and pre-mentalization), we first examined the configural invariance model in which factor loadings, intercepts, and residual variances were freely estimated. The configural invariance model had an acceptable fit [$\chi_{\left(136\right)}^{2}$ = 285.05, *p* < 0.001, CFI = 0.95, TLI = 0.94, SRMR = 0.09, RMSEA = 0.05], and was used as the baseline model for subsequent measurement invariance tests. Next, we examined the metric model, in which all loadings were constrained to be equal across groups. The metric model had a good fit [$\chi_{\left(160\right)}^{2}$ = 303.67, *p* < 0.001, CFI = 0.95, TLI = 0.95, SRMR = 0.08, RMSEA = 0.06], and the change in CFI and RMSEA were both < 0.01 (ΔCFI = 0.0, ΔRMSEA = 0.0). After establishing metric invariance, we examined the scalar model that constrained intercept to be the same across groups. However, the change in CFI was >0.01, and to address this, we released the constraints on three intercepts to establish partial scalar invariance. The partial scalar model yielded good model fit [$\chi_{\left(178\right)}^{2}$ = 340.76, *p* < 0.001, CFI = 0.95, TLI = 0.95, SRMR = 0.09, RMSEA = 0.07], and the change in CFI and RMSEA were both < 0.01 (ΔCFI = 0.01, ΔRMSEA = 0.01). As such, the partial scalar invariance model was established across the four groups.

**Table 3:** Fit indices for measurement invariance models.

**Models**	**χ^2^ (*df*)**	**CFI**	**ΔCFI**	**TLI**	**RMSEA**	**ΔRMSEA**	**SRMR**
**Continuous variables**							
Configural model	285.05 (*136*)^***^	0.95	—	0.94	0.09	—	0.05
Metric model	303.67 (*160*)^***^	0.95	0	0.95	0.08	0	0.06
Scalar model	391.06 (*184*)^***^	0.936	0.014	0.94	0.09	0.01	0.08
Partial scalar model	340.76 (*178*)^***^	0.95	0.01	0.95	0.09	0.01	0.07
**Categorical variables**							
Configural model	97.35 (*76*)^***^	0.99	—	0.99	0.05	—	0.06
Metric-scalar model	120.63 (*88*)^*^	0.99	0	0.99	0.05	0	0.06
Strict model	153.54 (112)^***^	0.99	0	0.99	0.05	0	0.08

Continuous variables consist of military PTSD and pre-mentalization. Continuous measurement invariance uses robust maximum likelihood. Categorical variables include corporal punishment and psychological aggression. Categorical measurement invariance use WLSMV. CFI, Comparative Fit Index; TLI, Tucker-Lewis Index; RMSEA, Root Mean Squared Error of Approximation; SRMR, Standardized Root Mean Squared Residual. Partial Scalar Model released constrained of 2 intercepts. ^*^p < 0.05, ^***^p < 0.001.

A slightly different approach was applied for the categorical variables (i.e., psychological aggression and corporal punishment). We started with a configural model that yielded good model fit [$\chi_{\left(76\right)}^{2}$ = 97.35, *p* < 0.001, CFI = 0.99, TLI = 0.99, SRMR = 0.05, RMSEA = 0.06]. Then we assessed metric-scalar invariance together in one model based on recommendations by Wu and Estabrook (2016). This model had good model fit [$\chi_{\left(88\right)}^{2}$ = 120.63, *p* < 0.001, CFI = 0.99, TLI = 0.99, SRMR = 0.05, RMSEA = 0.06], and the change in CFI and RMSEA were both < 0.01 (ΔCFI = 0.0, ΔRMSEA = 0.0). As a final step, we tested the strict invariance model, which constrained residuals to 1 for all latent variables over time. The strict model suggested good model fit [$\chi_{\left(112\right)}^{2}$ = 153.54, *p* < 0.001, CFI = 0.99, TLI = 0.99, SRMR = 0.05, RMSEA = 0.08], and the change in CFI and RMSEA were both < 0.01 (ΔCFI = 0.0, ΔRMSEA = 0.0).

### Group analysis

**Figure 3:**
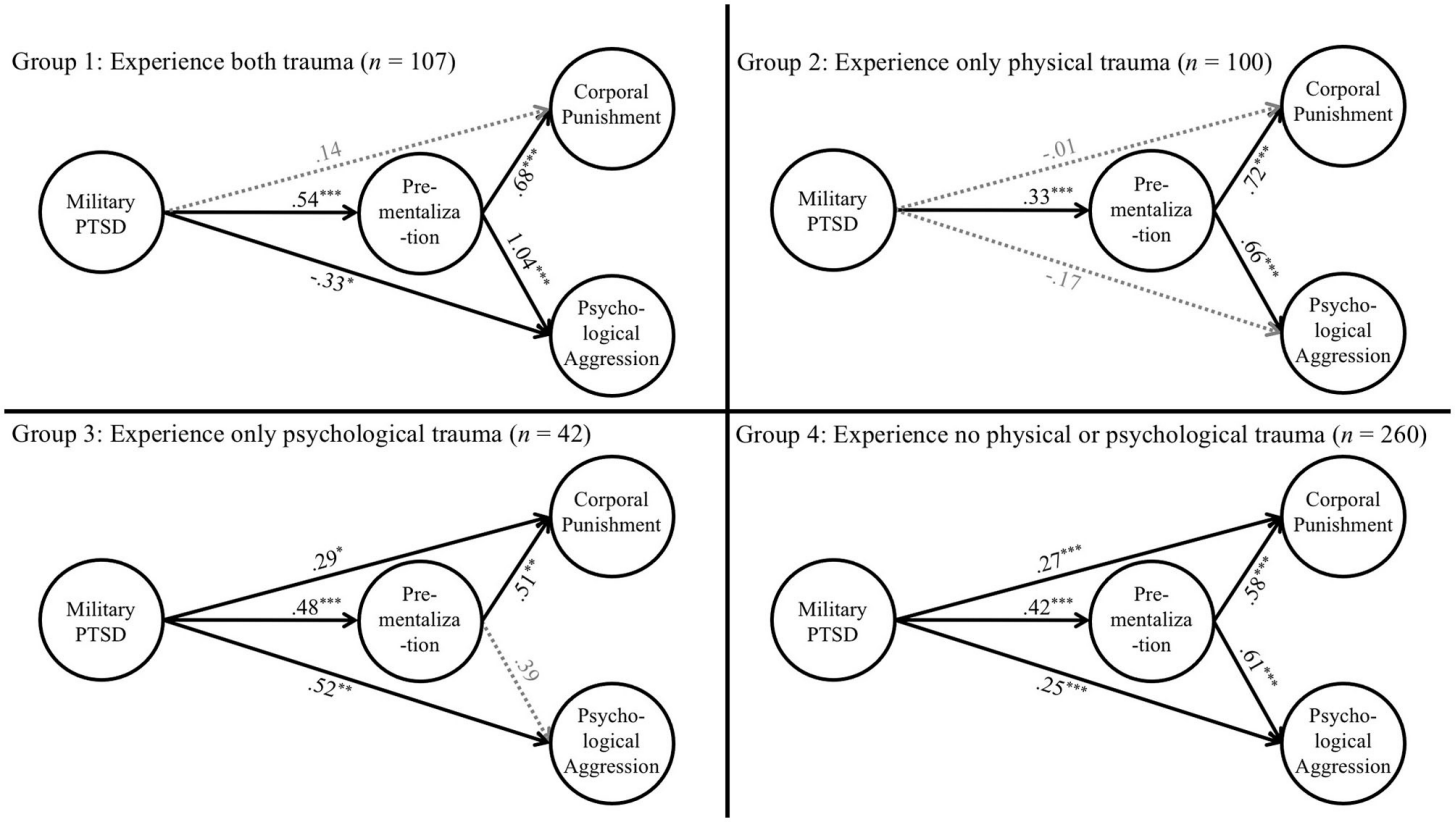
Group analysis. Group 1: Experience both trauma (*n* = 107). Group 2: Experience only physical trauma (*n* = 100). Group 3: Experience only psychological trauma (*n* = 42). Group 4: Experience no physical or psychological trauma (*n* = 260). *p < 0.05. **p < 0.01. ***p < 0.001.

After establishing measurement invariance for each latent variable, we fit our structural model and examined group differences by comparing the magnitude of each association across the four groups. The four groups included experiencing pre-military physical and psychological trauma (*n* = 107), experiencing only pre-military physical trauma (*n* = 100), experiencing only pre-military psychological trauma (*n* = 42), and experiencing no pre-military physical or psychological trauma (*n* = 260). Wald Tests were used to compare whether the magnitude of all effects as a group was significantly larger in some groups compared to others. As shown in Table 4 and Figure 3, a total of six group comparisons were conducted; however, only experiencing pre-military physical abuse significantly differed from experiencing no pre-military physical or psychological trauma (Wald = 16.86, *p* = 0.004).

**Table 4:** Group analysis of structural model.

	**Both trauma**	**Physical trauma**	**Psychological trauma**	**No physical and psychological trauma**
	***B*** **(*****SE*****)**	***B*** **(*****SE*****)**	***B*** **(*****SE*****)**	***B*** **(*****SE*****)**
Military PTSD -> Corporal punishment	0.14 (0.10)	−0.01 (0.08)	0.29 (0.14)^*^	0.27 (0.05)^***^
Military PTSD -> Psychological aggression	−0.33 (0.14)^*^	−0.17 (0.10)	0.52 (0.19)^**^	0.25 (0.06)^***^
Military PTSD -> Pre-mentalization	0.54 (0.06)^***^	0.33 (0.08)^***^	0.48 (0.11)^***^	0.42 (0.05)^***^
Pre-mentalization -> Corporal punishment	0.68 (0.11)^***^	0.72 (0.08)^***^	0.51 (0.17)^**^	0.58 (0.06)^***^
Pre-mentalization -> Psychological aggression	1.04 (0.13)^***^	0.66 (0.09)^***^	0.39 (0.22)	0.61 (0.06)^***^
**Residual variance**				
Military PTSD	0.93 (0.05)^***^	0.99 (0.01)^***^	0.98 (0.04)^***^	0.97 (0.02)^***^
Pre-mentalization	0.40 (0.07)^***^	0.82 (0.07)^***^	0.67 (0.12)^***^	0.69 (0.05)^***^
Psychological aggression	0.24 (0.06)^***^	0.30 (0.07)^***^	0.43 (0.13)^**^	0.37 (0.05)^**^
Corporal punishment	0.27 (0.08)^***^	0.50 (0.09)^***^	0.29 (0.15)^*^	0.36 (0.06)^*^
**Fit statistics**		
*χ^2^*	820.12			
*df*	706			
CFI	0.97			
TLI	0.96			
RMSEA	0.04			
SRMR	0.11			

Estimates shown are standardized coefficients. Estimates for parental race and poverty status are regressed on all latent variables in structural model. df, degree of freedom; CFI, Comparative Fit Index; TLI, Tucker-Lewis Index; RMSEA, Root Mean Squared Error of Approximation; SRMR, Standardized Root Mean Squared Residual. ^*^p < 0.05. ^**^p < 0.01. ^***^p < 0.001.

To identify which specific paths were significantly different between the two groups, we examined each of the paths separately. Figure 3 (Group 2 & 4) revealed that out of the five path comparisons, the effect of experiencing PTSD on psychological aggression, corporal punishment, and pre-mentalization, respectively, were all significantly higher for individuals in the group who experienced no pre-military physical or psychological trauma (compared to those experiencing only pre-military physical trauma).

### Indirect effects

As a final step, we formally tested the indirect effect of pre-mentalization as a potential mechanism across the four groups. There were significant indirect effects of pre-mentalization between military PTSD and corporal punishment for the groups that experienced pre-military physical and psychological trauma [*b* = 0.49, S.E. = 0.13, *p* < 0.001, 95% (0.23, 0.75)], experienced physical trauma [*b* = 0.29, S.E. = 0.10, *p* = 0.002, 95% (0.11, 0.48)], and no physical or psychological trauma [*b* = 0.41, S.E. = 0.10, *p* < 0.001, 95% (0.22, 0.60)]. In addition, there were significant indirect effects of pre-mentalization between military PTSD and psychological aggression for the groups that experienced pre-military physical and psychological trauma [*b* = 0.38, S.E. = 0.10 *p* < 0.001, 95% (0.18, 0.59)], experienced physical trauma [*b* = 0.12, S.E. = 0.04, *p* = 0.005, 95% (0.04, 0.20)], and no physical or psychological trauma [*b* = 0.14, S.E. = 0.03, *p* < 0.001, 95% (0.08, 0.20)].

## Discussion

Based on an online sample of 509 veteran parents, this study (1) used structural equation modeling to investigate the indirect effect of parental pre-mentalization between the association of veteran parents' military-related PTSD and harsh parenting, and (2) conducted multi-group analysis to determine if traumatic experiences of veteran parents prior to military service moderated the associations between parental military-related PTSD, pre-mentalization, and harsh parenting.

Our results suggest that pre-mentalization was a significant indirect effect of the association between military-related PTSD and harsh parenting, which supported our hypothesis based on mentalization theories. Specifically, veteran parents exhibiting higher PTSD symptoms were more inclined to employ pre-mentalization (i.e., interpreting their children's intention as negative), which in turn predisposed them to harsh parenting behaviors, such as corporal punishment and psychological aggression. This trend aligns with previous findings from other demographics, underscoring pre-mentalization as a critical factor in parent-child interactions (Dieleman et al., 2020; Yule, 2021; Edler et al., 2022). For example, Dieleman et al. (2020) showed that parents' pre-mentalization mediated the relationship between self-critical perfectionism and psychologically controlling parenting of adolescents.

Group analysis revealed intriguing patterns. The relationship between military-related PTSD and pre-mentalization was attenuated for parents who had only pre-military physical trauma compared to those without pre-military physical or psychological trauma. Additionally, there was an absence of a direct link between military PTSD and harsh parenting for parents who had only pre-military physical trauma. These findings lend some credence to the posttraumatic growth framework. We speculate that pre-military physical trauma may have equipped parents with coping mechanisms or fostered a transformed perspective on physical violence, particularly given their military experiences. Such adaptations might buffer the influence of PTSD symptoms on their parenting behaviors. This observed posttraumatic growth among veteran parents with a history of pre-military physical trauma resonates with existing literature on veterans, though prior studies primarily emphasized growth following military trauma or during the COVID-19 pandemic (Pietrzak et al., 2010; Tsai et al., 2015; Angel, 2016).

Our results did not show significant differences between groups of parents who experienced pre-military psychological trauma, parents who faced both pre-military physical and psychological trauma, and parents who had no pre-military physical or psychological trauma. This underscores the distinct interplay between types of pre-military trauma and military-related PTSD in veteran parents. While parallel research on the veteran population remains scant, our findings are somewhat consistent with studies that investigate the differential impacts of maltreatment types on PTSD. For instance, the association between psychological abuse and PTSD symptoms were more intense (Taft et al., 2005), which can be attributed to the oppressively fear-inducing environment established by the psychological abuser that profoundly undermines the victim's self-concept (Taft et al., 2008). Therefore, compared to their counterparts experiencing only pre-military physical trauma, people with psychological trauma may face more challenges in developing growth toward their PTSD symptoms. Nevertheless, these conclusions should be approached with caution, considering that the non-significance results could be caused by the limited sample size for each trauma subgroup, especially those who experienced only pre-military psychological trauma (*N* = 42).

### Limitations

Several limitations of our study warrant attention. Firstly, given our cross-sectional design, our results can only suggest associations rather than causations. Although our model is firmly rooted in theoretical and empirical foundations that guarantee reliable interpretations, future studies could employ a longitudinal approach to better understand causative relationships. Secondly, the reliance on self-reported measures for harsh parenting raises concerns about potential underreporting due to social desirability biases. Research involving children of veteran parents could provide a more holistic view. Additionally, self-reported measure on pre-mentalization may also not objectively represent participants' level of mentalization. As accumulating studies suggest main cortical areas involved in mentalization (Monticelli et al., 2021), future studies may consider using tools developed by neuroscience, such as functional magnetic resonance imaging (fMRI), to measure mentalization. Thirdly, our online data collection method might have inadvertently excluded those without internet or computer access, potentially skewing results. Lastly, with our sample being predominantly White males, the experiences of racial/ethnic minorities might be underrepresented. Nonetheless, our study sheds light on the seldom explored topic of harsh parenting by veteran fathers, offering a valuable contribution to parenting research on fathers.

### Strengths

While our study has certain limitations, it also has strengths. Our research adds innovations to parenting studies by examining the intricate interplay between PTSD, pre-mentalization, and harsh parenting within the understudied veteran population. Additionally, we revealed the profound intergenerational impacts of trauma by showing influences of parents' past traumas on the association between parental PTSD, pre-mentalization, and harsh parenting. We suggest the potential posttraumatic growth of veteran parents who experienced pre-military physical trauma. While contemporary research on parental mentalization has been largely mother-centric (Charpentier Mora et al., 2023), our study, with its focus on veteran parents, underscores the vital role of paternal mentalization in the realm of parenting.

## Implications

### Implications for clinical practice

The research findings offer insights for interventions with veteran families. While certain veteran parents with pre-military physical trauma demonstrated posttraumatic growth, the general trend indicates that PTSD may influence a propensity toward harsh parenting via pre-mentalization. As such, mentalization-based programs offer a promising approach for intervention, which aims to enhance individuals' ability to perceive and interpret the mental states of themselves and others. Effective in addressing a range of mental health conditions, from PTSD to personality disorders (Bateman and Fonagy, 2012), recent adaptations of these programs have focused on strengthening parental mentalization to foster positive parent-child interactions (Pajulo et al., 2006; Suchman et al., 2006, 2012; Slade et al., 2020; Barlow et al., 2021). Such interventions explored parents' attachment histories, beliefs, and emotions, empowering them to identify and manage theirs and their child's emotional states. The efficacy of these programs is evidenced by programs like the Mother and Toddler Program (MTP) by Suchman et al. (2006), which resulted in enhanced caregiving behaviors and improved maternal-child relationships among substance-using mothers. Similarly, the “Minding the Baby” (MTB) program, tailored for at-risk mothers under 25, not only improved maternal mentalization skills but also fostered secure attachments in their children compared to matched controls (Slade et al., 2020).

Considering that mentalization-based programs are predominantly developed for mothers, it's imperative to recognize the need for and potential benefits of such programs tailored to veteran fathers. Given that veteran fathers might constitute a significant portion of those utilizing these services, feasibility studies specifically focusing on this demographic are crucial. Adaptations suited to the unique experiences and challenges faced by veteran fathers will enhance the effectiveness and uptake of such interventions.

Secondly, we advocate for integrating more family-centric interventions within V.A. services, especially those emphasizing the intricacies of veteran family parent-child dynamics. While the V.A. acknowledges the pivotal role family members play in a veteran's recovery journey, the wellbeing and holistic development of military children should be accorded equal priority. The vulnerabilities of veteran parents, rooted in military-related PTSD and disproportionately high pre-military traumas, can inadvertently cascade to their children through suboptimal parenting practices. Tailored interventions for these families can identify and mitigate the potential repercussions of a veteran's traumatic experiences, thereby break the cycle of generational trauma. Additionally, by integrating the framework of posttraumatic growth into intervention designs, service providers can develop empowering programs that resonate with military culture.

### Implications for research

Existing research on veterans predominantly focus on the negative consequences of trauma, such as PTSD; however, it's equally important to understand positive changes that can arise after trauma. Our study suggests the potential of posttraumatic growth of veterans, while more studies are needed to reveal the mechanisms and factors that contribute to posttraumatic growth among veterans, so as to better help develop interventions. Finally, future studies could be devoted to examining the experiences of children in military households and elucidating the pathways underlying the intergenerational transmission of trauma and resilience.

## Conclusion

Our research highlights the indirect effect of veteran parents' pre-mentalization in the pathway between military-related PTSD and harsh parenting. Furthermore, our findings underscore the complex interplay between a parent's pre-military trauma, military-related PTSD, and harsh parenting. We advocate for the V.A. to integrate more family-centric interventions, specifically those centered on mentalization, to enhance parent-child dynamics, especially in families where parents suffer from military-related PTSD and pre-service trauma.

## Data availability statement

The raw data supporting the conclusions of this article will be made available by the authors, without undue reservation.

## Ethics statement

The studies involving humans were approved by Syracuse University Institutional Review Board. Written informed consent was not required in accordance with the local legislation and institutional requirements.

## Author contributions

XW: Conceptualization, Data curation, Formal analysis, Funding acquisition, Methodology, Project administration, Supervision, Validation, Visualization, Writing – original draft, Writing – review & editing. QL: Formal analysis, Methodology, Writing – original draft, Writing – review & editing. GM: Formal analysis, Methodology, Supervision, Writing – review & editing. AK: Writing – original draft. DY: Conceptualization, Writing – review & editing. AH: Writing – original draft.
